# The impact on healthcare facilities of the 2024 IV fluids shortage after Hurricane Helene: A mixed methods study

**DOI:** 10.1371/journal.pone.0344524

**Published:** 2026-04-09

**Authors:** Sophia Gorgens, Ahmad Alshadad, Almas Malek, Amit Boukai, Lindsay Davis, Attila J. Hertelendy, Fadi Issa, Christina Woodward, Amalia Voskanyan, Gregory Ciottone

**Affiliations:** 1 Department of Emergency Medicine, Yale University, New Haven, Connecticut, United States of America; 2 BIDMC Disaster Medicine Fellowship, Department of Emergency Medicine, Beth Israel Deaconess Medical Center and Harvard Medical School, Boston, Massachusetts United States of America; 3 Department of Information Systems and Business Analytics, College of Business, Florida, United States of America; Memorial Sloan Kettering Cancer Center, UNITED STATES OF AMERICA

## Abstract

**Background:**

In September 2024, Hurricane Helene damaged the Baxter International Factory in North Carolina as well as the surrounding infrastructure, causing the factory to shut down production. This resulted in a nationwide shortage of intravenous (IV) fluids. This study sought to characterize the severity of the shortage and define the problems hospitals faced.

**Methods:**

The National Critical Care Task Force is composed of 200 subject experts in hospital logistics and operations. A mixed methods approach was utilized, gathering quantitative data through an electronic REDCap survey and qualitative data through 1:1 semi-structured interviews. Descriptive summaries, frequencies, and proportions were produced from the quantitative survey data. The interview transcripts underwent thematic analysis using Braun & Clarke methodology.

**Results:**

Of the 200 critical care task force members, 17 participated in the survey (8.5%). 70.6% worked in urban hospitals and most (88.2%) were affected by the shortage. Hospitals created protocols to minimize IV fluids usage, including cancelling non-urgent surgeries. 11.8% hospitals asked for IV fluids from a neighboring hospital while 17.6% gave IV fluids to a neighboring hospital. 6 task force members participated in semi-structured interview. We identified six major themes: 1) Preparedness, 2) Impact of Shortage, 3) Response and Solutions, 4) Ethics and Resource Allocation, 5) Collaboration, 6) Governance.

**Conclusion:**

The impact of the IV fluids shortage after Hurricane Helene speaks to the fragility of the current system. This is the first study of its kind to delve into an analysis of the IV fluids shortage and to seek a framework for solutions.

## Introduction

In late September 2024, Hurricane Helene damaged the Baxter International Factory in Marion, North Carolina as well as the surrounding roads and infrastructure, causing the Baxter factory to shut down production [1,2]. This factory supplied a large portion of the United States’ intravenous (IV) fluids supply before the hurricane struck [3].Due to Hurricane Helene, all shipments from Baxter were canceled [1,2]. However, starting in early October, the company resumed limited operations and began to ship supplies to hospitals across the country in a reduced capacity [3]. The company allocated shipments to hospitals based on each hospital’s historical ordering and medical necessity.[1]. Many IV solutions, including Sodium Chloride 0.9% IV Solution, were previously considered to be in short supply, which was then further compounded by Hurricane Helene [4]

One survey found that “17 percent of providers were starting to cancel elective procedures and... 54 percent of providers... had 10 days or fewer of IV fluids in inventory.” [5]. The shortages were so severe that the Food and Drug Administration (FDA) temporarily approved imports of IV fluids from other countries [4,5] however, this was not the first time the United States faced medication shortages due to a hurricane [6]. When Hurricane Maria hit Puerto Rico in 2017, it caused damage to local infrastructure and factories, including 100 pharmaceutical and medical device manufacturers [6,7]. Of those damaged facilities, three factories were Baxter IV fluids factories, causing nationwide IV fluids shortages [6,7].

Shortages of critical supplies and medications can cause changes in standards of care and canceling of non-urgent or elective procedures [5,8,9]. IV fluids are a vitally important resource given their ubiquity and widespread use in all facets of medicine, from routine rehydration to life-saving treatments like dialysis [6]. While hospitals have come up with ad hoc solutions like using oral rather than IV medications whenever possible [10], published data is scarce, leaving each hospital to manage its own crisis [9,11]. Additionally, no national protocols exist for guidance.

This pilot study sought to characterize and define the problems hospitals face due to the IV fluids shortage and to elucidate current solutions and workarounds hospitals created. Specifically, its goal was to gather qualitative and quantitative data from hospitals across the United States on the impact of the shortage on patient care and clinical practice. Recognizing that resources and supply chains vary according to healthcare system size and location, the study sought to uncover operational instabilities and their solutions. While this analysis is the first to systematically quantify Hurricane Helene’s cascading effects on IV fluid availability across multiple healthcare tiers, it also addressed concerns about policymaking, equity in resource distribution, and the associated ethical implications. When there is a lack of policy for redistribution of production capacity geographically, the risk of supply chain disruption secondary to a natural disaster becomes much greater – which directly increases the need for mitigation and preparedness. Moreover, while well-resourced healthcare systems may adapt deftly to such shortages, under-resourced facilities may find themselves at a disadvantage where supply exhaustion rather than patient acuity may have to take precedence. From pandemics to hurricanes, natural disasters will continue to impact communities across the globe, with evidence pointing towards an increase in natural disasters over the coming years due to climate change [12]. Therefore, it is more important than ever to understand the fragility of supply chain logistics and the problematic nature of supply shortages within the healthcare system. Understanding how different healthcare systems have dealt with the 2024 IV fluids shortage will drive innovation for future preparedness and mitigation of similar nationwide disaster-driven supply shortages [3].

## Methods

To better understand and characterize the experiences of hospitals across the United States during the IV fluid shortage, a mixed methods approach with convergent design was utilized, gathering quantitative data through an electronic survey and qualitative data through 1:1 semi-structured interviews. The data was gathered sequentially in that within the quantitative survey, there was a possibility to opt into the qualitative interviews. However, the interview guide was pre-written and not influenced by the results of the survey. The results of both components of the study were statistically analyzed separately before integrating them for discussion. The purpose of the mixed methods design was to get a multifaceted and comprehensive understanding about the shortage. The Beth Israel Deaconess Medical Center Institutional Review Board reviewed and approved the study under exempt status.

### Study sample

The Taskforce for Mass Critical Care (https://masscriticalcaretaskforce.com) is composed of a group of experts who are primarily physicians in hospital logistics and operations [13]. There are about 200 members in this task force from varying medical backgrounds across the United States. All 200 members were contacted for both the quantitative and qualitative parts of this study. Those interested in participation were directed to use a link to a secure online data capture platform, REDCap, for eligibility screening. Eligibility criteria included being a member of the task force and age greater than 18 years. They were then directed to an online written informed consent form for electronic signature. The recruitment period for this study was March 10 – April 10, 2025, and data was gathered until May 31, 2025.

### Survey

Each participant was prompted to complete a 10-minute anonymous REDCap survey to gather data on personal and hospital demographics as well as information regarding the severity and impact of the IV fluids shortage. Researchers developed this survey instrument based on preliminary literature review and background research to capture key variables identified as relevant to the shortage.

*1:1 Semi-Structured Interviews*

For participants who opted into the 1:1 semi-structured interviews, a link to an encrypted Microsoft Teams Meeting was sent. These interviews were audio and visual recorded for the purposes of transcription and thematic analysis. Interviewers were members of the research team. An interview guide created by the research team served to facilitate discussion. Questions were generally open-ended and aimed to elaborate on aspects of the quantitative survey. Questions centered on preparedness, response, and recovery of healthcare systems during the IV fluids shortage as well as reflections on future improvements. The session lasted 45–70 minutes. After completion of the interview, the written transcript was de-identified by the lead author for analysis and the video recording was deleted.

The interview guideline included:

- Discussion of the 2024 IV fluids shortage such as challenges faced by the participant’s healthcare system, solutions proposed, effective vs ineffective solutions, hospital’s overall preparedness
- Discussion of prior supply chain shortages such as if the participant’s healthcare system was affected by Hurricane Maria in 2017, other pharmaceutical or medical supply shortages they experienced in their career, problems and solutions encountered during these prior shortages, and if the lessons learned from these prior situations guided or influenced the response to the 2024 IV fluids shortage.
- Discussion of best practices and future preparedness including their own opinions or the protocols or strategies of their healthcare system, the applicability of such guidelines nationally, the role of mutual aid or relationships, the role of government (if any) in providing aid during a shortage, and the ethics of IV fluids restrictions or changes in clinical practice secondary to the shortage.
- Open forum for any additional comments.

### Quantitative analysis

Descriptive summaries were produced to assess all variables and to describe the study sample. Frequencies and proportions were calculated for questions such as those regarding days of IV fluids supply, methods of conservation, and reduction of IV fluids use.

### Qualitative analysis

The interview transcripts were analyzed using Braun & Clarke methodolog [14]. Members of the research team trained in qualitative analysis reviewed the data. Coding was done manually by a minimum of two researchers. Braun & Clarke methodology is a 6-step method of thematically assessing the data that is flexible but context-rich [14]. In step 1, the reviewers familiarize themselves with the data. For step 2, each reviewer generates initial codes about the data to help organize it into meaningful groups. Step 3 focuses on the creation of broader themes observed in the data. In step 4, themes are reviewed and refined to make a thematic map for the data. For step 5, the themes continue to be refined and then applied to the data to analyze it. Part of the refining process includes comparing the coding and derived themes between all reviewers and reassessing when they do not match. In the final step, the data is written up into a coherent and logical report based on the data analysis.

Sample size for the qualitative portion was determined using the information power concept, which posits that studies require fewer participants when the research aim is narrow and the quality of dialogue is high. Given this study’s focused objective and the extensively detailed interviews obtained, six interviews provided adequate information power to address the research questions [15]. The criteria for the rigor and trustworthiness of the qualitative data included credibility, transferability, dependability, and confirmability [16,17]. Credibility was established by reflective listening and member checking during the interviews as well as by having a minimum of two researchers code the transcripts. Transferability was facilitated by detailed descriptions of the situations of the healthcare facilities and their clinical practices. Dependability was ensured by a mandatory review session on coding and analysis as well as by consistent and documented coding decisions. Confirmability was enabled by reflexive discussions on bias during the review session and well-maintained data showing the links between the raw data and codes.

## Results

### Quantitative data

Of the 200 critical care task force members, 17 participated in the survey (8.5%) (supporting document: survey results). The median participant age was 44 years (IQR 40, 51), predominately male (n = 12, 70.6%), and predominately medical doctors (n = 15, 88.2%). Most participants worked in critical care (n = 13, 76.5%) with a primary role as a clinician (n = 10, 58.8%), although 35.3% served as clinical directors. They had an average of 18 years of experience in healthcare (IQR 14, 25) and 10 years in their current position (IQR 5, 19).

The majority of participants worked in urban hospitals (n = 12, 70.6%), with suburban (n = 3, 17.6%) and rural (n = 2, 11.8%) also represented. 76.5% of the hospitals had a capacity of more than 250 beds. 82.4% (n = 14) were part of a larger system and 64.7% (n = 11) had 6 or more hospitals in their system.

Most hospitals (n = 15, 88.2%) were affected by the IV fluids shortage. Before the shortage, no hospitals had less than a 3-day supply of IV fluids stocked and some (n = 2, 11.8%) had more than a 30-day supply; however, during the shortage this changed to 11.8% (n = 2) with less than a 3-day supply and none with more than a 30-day supply. No hospitals ran out of IV fluids completely. 64.7% (n = 11) of hospitals had to limit which patients were eligible to receive fluids during the crisis. Patient populations that still received IV fluids included sepsis (n = 11, 64.7%), septic shock (n = 13, 76.5%), any type of clinical shock (n = 14, 82.4%), and as per clinical judgement (n = 11, 64.7%). Fluid restriction measures included switching IV medications to oral (n = 13, 81.3%), giving IV medications as push doses rather than in fluids (n = 10, 62.5%), and restricting IV hydration (n = 13, 81.3%). 58.8% (n = 10) of hospitals created a protocol for oral hydration, including water, soda, juice, and electrolyte drinks. Although 29.4% (n = 5) of hospitals had to cancel elective surgical procedures, none canceled urgent or emergent surgical procedures. Additional solutions and work arounds were also described (Table 1). All of these conservation methods helped hospitals decrease their IV fluids usage by an average of 40% (IQR 25, 55).

**Table 1:** Quotes from participants survey open-ended responses for additional solutions used during the recent IV fluids shortage and suggestions for future preparedness.

Describe any additional solutions your hospital found effective
“Targeted educational and outreach activities; increased antimicrobial stewardship”
“EMR BPA but it was often brushed aside especially in the ICU. Small fluid bags were not as easy to obtain and therefore larger bags were spiked. CRRT was also a major usage of IV fluids”
“Increased use of oral agents and discouragement of maintenance fluids sufficed.”
“Frequent evaluations by clinical provider leaders of utilization of IV fluids to calibrate conservation”
“Education of patients via signs, pamphlets and on the internal TV channel as to what and why”
“Changing NPO policies prior to minor procedures, working with anesthesia to place IVs but not routinely hang bags of fluids, choosing a ‘fluid of the week’ preference based on availability during any given week”
**Describe any suggestions you might have for hospitals to better prepare for and deal with future IV fluid shortages**
“Diversify supply chains, have contingency fluids accessible, preemptive mitigation and rapid response and recovery”
“Need multiple vendor/ production sites in the US”
“Dual sourcing, changing clinical practices, reserving fluids for those truly needing it with certain criteria and having smaller amounts available”
“Maintenance fluids are usually a bad idea, and being forced to avoid them was perhaps an improvement in care”
“Push industry to put a significant plant somewhere far from the coast or other major weather disaster areas (not tornado alley either)”
Diversify suppliers

During the shortage, 11.8% (n = 2) hospitals had to ask for additional IV fluids from a neighboring hospital while 17.6% (n = 3) had to give additional IV fluids to a neighboring hospital. While the majority (n = 7, 41.2%) of hospitals were affected by the shortage between 2 and 3 months, 23.5% (n = 4) still had ongoing issues as of March 2025 (over 6 months later).

In terms of preparedness, 100% (n = 17) of hospitals include supply shortages in their emergency operation plan (EOP) but only 41.2% (n = 7) specifically include IV fluids shortages. About half of hospitals (n = 9, 52.9%) had backup vendors for IV fluids but while 81.8% (n = 9) of these were able to get some additional fluids, none got enough to end their hospitals shortage and one hospital (9.1%) was unable to get any additional fluids from their backup vendor. During the last nationwide IV fluids shortage, which was caused by Hurricane Maria in 2017, 35.3% of hospitals were affected and created fluid conservation methods at that time.

### Qualitative data

Of the 17 who completed the survey, 6 participated in a 1:1 semi-structured interview. We identified six major themes: 1) Preparedness, 2) Impact of the Shortage, 3) Response and Solutions, 4) Ethics and Resource Allocation, 5) Collaboration, 6) Governance.

#### Theme 1: Preparedness.

Within the disaster management cycle, preparedness refers to planning activities that increase the readiness of healthcare systems for when disasters occur with the goal of decreasing their impact. Participants discussed ways in which their healthcare systems were prepared to handle the IV fluids shortage, including increased experience with a variety of shortages, protocol adaptability, and more effective systems for tracking utilization and remaining reserve of supplies. One person noted, “I think we’ve gotten better at adapting protocols. And adapting practices to account for the possibility of shortages.” While the lessons-learned from prior shortages were not always codified in new protocols, most believed that the combination of living memory and clinician flexibility to adapt to new challenges were more than adequate to address the supply shortages as they arose.

Participants also discussed failure points in preparedness, such as the difficulties of stockpiling perishable items and just-in-time supply chains. One participant in particular spoke to the difficulties of a supply-and-demand economy: “You know the corporatization of medicine in America, right? I mean, it’s just in time purchasing. You know we can’t get these guys to plan more than 30 seconds in advance.” Their point was that the economic incentives for disaster preparedness such as stockpiling or robust supply chain logistics are often not visible or easily grasped. Disaster preparedness pays dividends in the future – but only if a disaster occurs.

Lastly, participants provided recommendations for bolstering future preparedness by reevaluating clinical practices and suggesting private-public partnership and redundancy plans.

#### Theme 2: Impact of the Shortage.

While some respondents noted a minimal impact on their healthcare systems and others noted a severe impact, it was universally acknowledged that unlike other pharmaceutical or medical supplies shortages that were regionalized or system-specific, the IV fluids shortage was ubiquitous. Its effect was both negative and positive. Negative impacts included threats to patient care and safety, such as having limited resources to treat patients as one usually would. The most common cited example was sepsis, where if IV fluids are withheld in certain clinical scenarios the situation could become life-threatening. One participant captured this by stating, ““The main challenge was… discrepancies between what I felt was the standard of care. The main thing that comes to mind is sepsis… and trying to figure out if am I able to decrease the amount of fluids, or is this an appropriate clinical scenario to actually give sepsis protocol fluids?”

Psychological stress to providers played a significant role in participants’ perspective of the shortage as well, with one person saying, “There was always that knowledge that we are in a national shortage in the background of my mind to make me kind of double think, should I be giving fluids to this person or not.” While they admitted this helped them be more thoughtful and considerate of their clinical practice, it also created a constant anxiety.

Positive impacts included decreased medical waste of IV fluids and judicious use of fluids resulting in improvements in care in certain cases. Additionally, many other shortages of medical and pharmaceutical supplies were discussed, along with their impacts. This included blood culture bottles (which resulted in a more judicious use of this product), IV contrast (causing an increase in non-contrast CT scans, which were not always effective), and medications such as piperacillin-tazobactam (resulting in increased antimicrobial stewardship).

#### Theme 3: Response and Solutions.

Within the disaster management cycle, response refers to the immediate actions during and in the acute phase after a disaster and generally includes emergency medical care, resource mobilization, and restoration of critical infrastructure and supplies. Throughout healthcare systems, response to the IV fluids shortage was varied but robust. This included alerts on the electronic medical record, daily meetings with key stakeholders, and open communication. The transparency of expectations and the teamwork between all levels of employees was instrumental to the success of many of these strategies. As a participant stated, “[The hospital] mandate was: we want you to have conversations with every one of your providers… in your service line, not just sending an e-mail. This is so important that it has to be a conversation, and you have to be having closed loop communication. [The hospital] set that expectation from the beginning.”

To help with redistribution and allocation of supplies, there was often a centralization of IV fluids in the pharmacy, reallocation of IV fluids from areas of low urgency, cancellation of elective procedures, oral hydration protocols, and extension of expiration dates. However, these solutions were not without their drawbacks. One participant noted that “Centralization [of IV fluids] is helpful... at the expense of delays in using that supply when needed.”

When reflecting on the effectiveness of what their healthcare systems implemented or untapped potential solutions, participants proposed that future shortages could be mitigated by having disaster plans and protocols in place, decreasing medical waste and increasing reusability of products, fostering regional hospital collaboration, and diversifying the supply chain.

#### Theme 4: Ethics and Resource Allocation.

A core tenet of disaster medicine is to do the most good for the most number of people in situations where patient needs overwhelm available resources. In such situations, altered standards or crisis standards of care might be considered. Disaster medicine therefore necessarily encompasses ethical deliberation alongside clinical and operational concerns. Participants of this study acknowledged that in a disaster situation such as a critical IV fluids shortage, alternative standards of care were, in some cases, implemented, even if they were not labeled as such by the healthcare system. For many participants, this included canceling non-emergency surgery cases. Although canceling elective surgeries might be doing a disservice to those patients whom it affects, it might be the only way to ensure there are enough IV fluids to treat patients with life-threatening conditions. Yet there is a potential for inequity in a fractured national healthcare system such as the United States has where hospitals often exist in silos, isolated from other hospitals. Specifically, one participant stated, “I worry about some hospitals having to cancel non-elective or semi-urgent admissions or procedures while other hospitals continue to like practice full tilt because they have access to these resources and don’t want to cancel elective cases, which are often revenue generating for the system”

While most participants were impressed with the collaboration between neighboring hospitals during this time of crisis, they did worry that, “Some of the smaller hospitals that didn’t have as many resources… tended to suffer more.” If larger healthcare systems have access to more resources, participants noted, it is ethically laudable to help neighboring hospitals under critical stress from the shortages.

#### Theme 5: Collaboration.

Collaboration can take many forms, from national or state mobilizations to formal mutual aid agreements to aid rendered due to sense of civic responsibility. Capturing this sentiment, one participant of the study stated, “I think [collaborative relationships are] something that every system needs to cultivate. Because if you don’t have those relationships when the times are good, it’s going to be very hard to form those relationships and have them be productive and fruitful when it’s a stressful situation.”

During the IV fluids shortage, success in handling the crisis was made possible through the partnership of hospital leadership and staff, from physicians to nurses to pharmacists. Working with staff to come up with responses and solutions increased camaraderie and support from these healthcare workers, which increased their effectiveness in implementation. While in some instances, collaboration between hospitals across the region was hindered by financial concerns, in other places collaboration thrived through civic duty and cultivation of these interhospital relationships before the IV fluids shortage. One participant observed, “A really unfortunate thing with healthcare is that we approach it more capitalistically. We approach it in a competitive way. And I think that that’s a disservice.” The consensus among participants appeared to be that while market forces of supply-and-demand may encourage competitive behavior that threatens to harm patients in times of crisis, the humanity of clinicians and administrators won out more often than not during the IV fluids shortage. Collaboration and aid among hospitals was the norm rather than the aberration.

#### Theme 6: Governance.

Within the field of disaster medicine, governance refers to the policy-making and existing legal infrastructure as well as the authority structure and systems coordination for dealing with all aspects of the disaster management cycle (mitigation, preparedness, response, and recovery). Participants of this study reflected on what roles, if any, government should take in the management of supply chain logistics, which differs from other disasters such as earthquakes or hurricanes themselves in that it may be less visible and less well defined. Still, they decided, many of the guiding principles remained the same: “For a nationwide disaster that impacts everybody like this IV fluid shortage, I think there has to be some sort of federal coordinating role.”

Given that most disasters are local or regional, response to a crisis (or at least augmentation of a hospital’s response, as needed) usually falls into the purview of state government. As one participant emphasized, “Having enough of [a regional or state] stockpile that if something happened to your hospital and you needed three days of critical supplies is hugely important].” However, the IV fluids shortage impacted healthcare systems across the country. While all praised the Strategic National Stockpile and its key role in safeguarding the healthcare system in many regards, one person noted “the federal level is more about what are the regulatory barriers and or incentives that make us consolidate our supply chain in such a narrow fashion” Federal policy, therefore, could be beneficial in guiding response and preparedness, especially in regulating or assisting the production and distribution of scarce resources.

## Discussion

Although healthcare systems in the United States were affected to varying degrees of severity, the impact from the IV fluids shortage after Hurricane Helene destroyed the Baxter factory was ubiquitous. While some healthcare systems were not extensively affected, almost all had to make changes and concessions to accommodate the sudden decrease in supply. This shortage highlighted the complexity of supply chain logistics as well as the economic incentives for just in time purchasing, hyper-specialization of production, and consolidation in medical supply production [18,19].

Although all hospitals had a supply shortage emergency operations plan (EOP) before Hurricane Helene, the consensus from participants was that while the organizations adapted quickly, there was no specific IV fluids shortage plan and many measures taken felt ad hoc. A lack of depth to hospital preparedness therefore resulted in a reactive response rather than a proactive defense.

Failure to be properly prepared for disasters can have undesirable and, at times, dire consequences. While the Joint Commission on Accreditation of Healthcare Organizations recommends hospitals be self-reliant up to 72 hours following an incident, during the 2024 IV fluids shortage two hospitals reportedly had less than 72 hours’ worth of IV fluids stored, putting them at risk of disrupted continuity of care had an incident occurred at that time [20]. Many hospitals also had to cancel elective surgeries. Cancelling elective cases carries negative implications such as the burden of rescheduling backlogged procedures once the shortage is over, loss of revenue, delaying diagnosis and treatment, and prolonging patients suffering. Moreover, in prolonged period of resource limitation can cause stewardship and decision-making fatigue for both administrators and clinicians [3].

Although approximately two thirds of the hospitals had to limit which patients received IV fluids during the shortage, participants did not recall this having a significant impact on clinical care. This could be indictive of rapid response measures from the hospitals as well as good clinical judgment. While having clear policies ahead of time offloads the ethical burden of difficult decision-making, during this IV fluids crisis, physicians appeared to adapt swiftly. However, even in times of plenty, it would behoove healthcare facilities to be cognizant of waste and inefficient use of resources [21].

Success in response to shortages within the hospital must come from an ability to achieve buy-in from all relevant parties, creating a unified environment to tackle the shortage. Having a team made up of key stakeholders monitoring the shortage allows the system to be responsive and dynamic. An average decrease in IV fluids consumption of 40% is a great achievement – put into perspective, a hospital with 3 days’ worth of IV fluids could potentially extend that period by about 28 hours. The successes of decreased IV fluids consumption was largely due to interdisciplinary teamwork.

Many believe that there is a civic duty for larger hospital networks to assist smaller hospitals in disasters. Interfacility collaboration and strategies such as patient-load balancing have been shown to be effective and efficient [22–25]. Our study revealed that regional collaboration between hospitals was utilized effectively, mainly due to well-maintained inter-hospital personal connections. However, while the collaboration of healthcare systems is vital to patient load-balancing and equitable care, the authors recommend that for all hospitals to have access to an adequate supply of critical medical supplies, the support of the state or federal government is desirable. The effectiveness of this strategy has already been demonstrated by the Strategic National Stockpile (SNS), but given the new medical supply shortages in recent years, expansion of the role of the SNS should be considered [26]. Moreover, although niche specialization in production may make economic sense, the authors further recommend that versatility and adaptability in production should be reviewed by policy makers to better mitigate both the short- and long-term effects of natural disasters on supply chains.

By revealing the challenges and deficiencies in preparedness, this study has laid the foundations for further work, which would include the creation of national guidelines and protocols to prepare for, mitigate, and respond to supply shortages at hospitals in the future. Natural disasters are expected to increase with climate change, and supply chains will continue to be affected by these, endangering hospitals and their communities.

### Limitations

This study was a pilot study and limited in scope. Most responses came from large urban health systems, and the results may therefore not be generalizable to rural and suburban communities. Although most of the respondents were intensive care or internal medicine physicians, many had with administrative roles with an overview of the entire system, which may have limited the sample bias; however, some specialties may have been overlooked in their insight. Additionally, the sample sizes for both qualitative and quantitative aspects of the study were small and the response rates were low which may limit generalizability.

## Conclusion

The ubiquity of the impact from the IV fluids shortage after Hurricane Helene destroyed the Baxter factory speaks to the fragility of the current system. This is the first proposed study of its kind to delve into an analysis of the IV fluids shortage and to seek out a framework of solutions. It revealed a lack of preparedness for the niche supply-chain logistics of medical supplies with specific expiration dates but an overall awareness and growing ability to respond to these recurring types of shortages. There is therefore a need for collaboration not only on a national or state level through policies but also on a community level between neighboring hospitals.

## Supporting information

S1 File Survey Results. This supporting document includes the data for the results of the survey sent out to participants, including participant demographics and specifics about the IV fluids shortage.
(DOCX)
