# Supplementary material for: The impact on healthcare facilities of the 2024 IV fluids shortage after Hurricane Helene: A mixed methods study
Source: PLoS One. 2026 Apr 9;21(4):e0344524. doi: 10.1371/journal.pone.0344524 (PMC13065003; doi:10.1371/journal.pone.0344524)
Supplement: S1 File — This supporting document includes the data for the results of the survey sent out to participants, including participant demographics and specifics about the IV fluids shortage. (DOCX) [file pone.0344524.s001.docx]

**Survey Results**

*This supporting document includes the data for the results of the survey sent out to participants, including participant demographics and specifics about the IV fluids shortage.*

Survey N=17, Interview N=6

Median age 44 years (IQR 40, 51)

Gender male (12, 70.6%)

Educational background MD (15, 88.2%), Nurse practitioner (2, 11.8%)

MD 12

Nurse practitioner 1

1 MD and PhD

2 MD and Master’s

1 NP and Master’s

Specialty Critical Care (13, 76.5%), Internal Medicine (4, 23.5%)

Years in healthcare median 18 (IQR 14, 25)

Primary role in hospital: clinician (10, 58.8%), clinical director (6, 35.3%), other (1, 5.9%)

Median years of experience in position: 10 (IQR 5, 19)

Hospital type

Rural (2, 11.8%), Suburban (3, 17.6%), Urban (12, 70.6%)

Is your hospital a part of a larger system?

Yes (14, 82.4%), No (3, 17.6%)

Do you have multiple hospitals within your system?

Solo (1, 5.9%), 2-5 hospitals (5, 29.4%), 6-10 hospitals (5, 29.4%), 11+ hospitals (6, 35.3%)

Hospital size:

< 40 beds (0, 0.0%), 41-100 beds (1, 5.9%), 101 - 250 (3, 17.6%), 250+ (13, 76.5%)

Was your hospital or the hospitals you represent affected by the fluid shortage?

Yes (15, 88.2%), No (2, 11.8%)

Did your hospital(s) limit which patients were eligible to receive fluids?

Yes (11, 64.7%), No (6, 35.3%)

Select all that apply for which population of patients received IV fluids:

Not applicable (i.e. your hospital had no restrictions) (3, 17.6%), Sepsis (not necessarily septic shock) (11, 64.7%), Septic shock (13, 76.5%), Any type of clinical shock (14, 82.4%), Clinical judgement (11, 64.7%)

Select all the fluid restriction measures your hospital used:

a. Switch some IV medications to oral (13, 81.3%), b. Give IV medications like ceftriaxone as push doses rather than in fluids (10, 62.5%), c. Restrict IV hydration (13, 81.3%), d. Other {measures_other} (3, 18.8%)

Did your system create a protocol for oral rehydration?

Yes (10, 58.8%), No (7, 41.2%)

What type of oral fluids were available? Select all that apply

a. Water (13, 86.7%), b. Soda (Cola, ginger ale, etc.) (8, 53.3%), c. Juice (9, 60.0%), d. Gatorade, pedialyte, or similar electrolyte drink (13, 86.7%)

Were elective surgical procedures cancelled?

Yes (5, 29.4%), No (12, 70.6%)

Were urgent or emergent surgical procedures cancelled?

Yes (0, 0.0%), No (17, 100.0%)

By what percentage did the hospital manage to decrease its IV fluid usage?

10% (3, 17.6%), 20% (2, 11.8%), 30% (3, 17.6%), 40% (2, 11.8%), 50% (3, 17.6%), 60% (3, 17.6%), 70% (1, 5.9%), 80% (0, 0.0%), 90% (0, 0.0%)

Before the shortage, how many days of IV fluids did your hospital typically stock?

1 day (0, 0.0%), 1-3 days (0, 0.0%), 3-10 days (3, 17.6%), 10-30 days (3, 17.6%), >30 days (2, 11.8%), not sure (9, 52.9%)

During the shortage, how many days of IV fluids stocked was the lowest that your hospital reached?

1 day (0, 0.0%), 1-3 days (2, 11.8%), 3-10 days (4, 23.5%), 10-30 days (3, 17.6%), >30 days (0, 0.0%), not sure (8, 47.1%)

Did your hospital ever run out of IV fluids completely

Yes (0, 0.0%), No (17, 100.0%)

Did your hospital have to ask for additional IV fluids from a neighboring hospital?

Yes (2, 11.8%), No (15, 88.2%)

Did your hospital have to give additional IV fluids to a neighboring hospital?

Yes (3, 17.6%), No (14, 82.4%)

How long did the IV fluid shortage affect your hospital?

Less than 1 week (1, 5.9%), Less than 1 month (2, 11.8%), Less than 2 months (3, 17.6%), Less than 3 months (7, 41.2%), Ongoing issues as of March 2025 (4, 23.5%)

Are supply shortages or supply chain logistics issues specifically discussed in your hospital's emergency operation plan (EOP)?

Yes (17, 100.0%), No (0, 0.0%)

Is IV fluid shortage specifically discussed in your hospital's EOP?

Yes (7, 41.2%), No (10, 58.8%)

Did your hospital have a backup vendor for IV fluids?

Yes (9, 52.9%), No (8, 47.1%)

If yes, was your hospital able to get IV fluids from this backup vendor?

None (1, 9.1%), Some (9, 81.8%), almost enough (1, 9.1%), Enough to not restrict IV fluids at the hospital (0, 0.0%), Enough to have the normal amount of IV fluids the hospital always has (0, 0.0%)

The last nationwide IV fluid shortage of similar proportions was Hurricane Maria. Was your hospital affected by that shortage?

Yes (6, 35.3%), No (4, 23.5%), Unsure (7, 41.2%)

If your hospital was affected by the IV fluid shortage from Hurricane Maria, please describe any steps the hospital took afterwards to prepare for or mitigate future shortages and their effectiveness:

1. Created dashboards to track IV fluid use. Created alternative order sets to reduce specific IV fluids. These were able to be turned back "on" during recent shortage
2. None
3. Switching most electrolytes to PO replacement
4. Similar to this one, oral when possible, and some IV infusions changed to push

Please describe any additional challenges your hospital might have faced:

1. Only a few very specific surgeries were cancelled due to shortage of irrigation fluids.
2. Provider and clinician educational gaps in approaches
3. Lack of sterile IV flushes

Please describe any additional solutions your hospital found effective:

1. Targeted educational and outreach activities; antimicrobial stewardship increased
2. Used EMR BPA but it was often brushed aside especially in the ICU. Small fluid bags were not as easy to obtain and therefore larger bags were spiked. CRRT was also a major usage of IV fluids
3. The actual impact on patient care was not extreme. Increased use of oral agents and discouragement of maintenance fluids sufficed.
4. Frequent evaluations by clinical provider leaders of utilization of IV fluids- daily review of utilization to calibrate conservation
5. Education of patients via signs, pamphlets and on the internal TV channel as to what and why
6. changing NPO policies prior to minor procedures, working with anesthesia to place IVs but not routinely hang bags of fluids. choosing a 'fluid of the week' preference based on availability during any given week

Please describe any suggestions you might have for hospitals to better prepare for and deal with future IV fluid shortages:

1. Diversify supply chains, have contingency fluids accessible; preemptive mitigation and rapid response and recovery
2. we need multiple vendor / production sites in the US
3. dual sourcing, changing clinical practices, reserving fluids for those truly needing it with certain criteria and having smaller amounts available
4. Maintenance fluids are usually a bad idea, and being forced to avoid them was perhaps an improvement in care.
5. They should push industry to put a significant plant somewhere far from the coast or other major weather disaster areas (not tornado alley either) like say Maine or Michigan, where there is lots of water and low disaster rates historically
6. diversify suppliers
